# Patients’ and staff members’ experiences of restrictive practices in acute mental health in-patient settings: systematic review and thematic synthesis

**DOI:** 10.1192/bjo.2022.574

**Published:** 2022-10-06

**Authors:** Hannah Butterworth, Lisa Wood, Sarah Rowe

**Affiliations:** Division of Psychiatry, University College London, UK; Division of Psychiatry, University College London, UK; and North East London Foundation Trust, Acute and Rehabilitation Directorate, Goodmayes Hospital, London, UK

**Keywords:** Systematic review, thematic synthesis, restrictive practices, acute mental health in-patient care, qualitative research

## Abstract

**Background:**

Recent guidance has called for the reduction of restrictive practice use owing to growing concerns over the harmful physical and psychological effects for both patients and staff. Despite concerns and efforts, these measures continue to be used regularly to manage challenging behaviour in psychiatric in-patient settings.

**Aims:**

To undertake a systematic review of patients’ and staff members’ experiences of restrictive practices in acute psychiatric in-patient settings.

**Method:**

A systematic review and thematic synthesis was conducted using data from 21 qualitative papers identified from a systematic search across three electronic databases (PsycInfo, Embase and MEDLINE) and citation searching. The protocol for the review was pre-registered on PROSPERO (CRD42020176859). The quality of included papers was examined using the Critical Appraisal Skills Programme (CASP).

**Results:**

Four overarching themes emerged from the experiences of patients: the psychological effects, staff communication, loss of human rights and making changes. Likewise, the analysis of staff data produced four themes: the need for restrictive practices, the psychological impact, decision-making and making changes. Patient and staff experiences of restrictive practices were overwhelmingly negative, and their use carried harmful physical and psychological consequences. Lack of support following restraint events was a problem for both groups.

**Conclusions:**

Future programmes seeking to improve or reduce restrictive practices should consider the provision of staff training covering behaviour management and de-escalation techniques, offering psychological support to both patients and staff, the importance of effective staff–patient communication and the availability of alternatives.

When it comes to acute psychiatric care, patients should be treated in the least restrictive environment possible.^1^ Internationally, in-patient psychiatric settings are reserved for high-risk individuals with the most severe and complex mental health problems, including schizophrenia and personality disorder.^2–4^ There is general consensus worldwide that the aim of psychiatric in-patient care should be to provide a safe, humane and therapeutic environment during acute episodes of mental illness.^5,6^ The rise in detainment rates under mental health legislation has meant that a larger proportion of in-patients are receiving care against their will, rather than agreeing to their admission.^7,8^ Under these circumstances, behaviours such as self-harm, suicide, violence and aggression can be common in acute psychiatric in-patient settings.^9^

In the management of high-risk behaviours, clinical guidelines recommend that staff employ a non-coercive method to de-escalate a situation.^10,11^ These may include verbal techniques, constant monitoring or reducing environmental risks.^12^ If these are ineffective, or if the situation calls for more urgent de-escalation, restrictive interventions may be implemented. Restrictive practices refer to a range of measures. Physical and chemical restraint (e.g. rapid tranquillisation) aim to restrict movement or control behaviour in an emergency,^12^ and seclusion is intended to isolate and reduce sensory stimulation to calm the patient and ensure everyone is safe from harm.^13^ In a wider sense, a patient's liberty may be restricted; for example, disallowing access to outdoor space.^14^ Restrictive practices should only be employed as a last resort.^1^ However, research suggests that these measures are still commonly used, sometimes excessively or unnecessarily.^1,15^ This is a problem worldwide despite a lack of evidence to back up their effectiveness.^4^ Over-reliance on restrictive techniques has been attributed to several challenges and pressures facing staff working in psychiatric in-patient settings, including a lack of effective alternatives,^12,16^ significant staff shortages and an insufficient number of professionals on in-patient teams who are trained in the de-escalation of challenging behaviour.^12,17^ As a consequence, reducing restrictive practices is a priority at international levels and a number of initiatives have been outlined to achieve this.^11^ In the UK, the Royal College of Psychiatrists has described a reducing restrictive practices programme that includes de-escalation strategies, whole service interventions and improving staff competence.^15^ However, despite such initiatives, restrictive practices continue to be an area of concern.

There is growing concern over the detrimental physical and psychological effects of restrictive practices.^18^ Current qualitative research suggests that restraint events can produce high levels of distress, fear and anxiety in both patients and staff.^12,19^ Restraint and seclusion can also cause re-traumatisation for those who have experienced physical or sexual abuse.^20^ Concerningly, a 2019 systematic review reported a post-traumatic stress disorder (PTSD) rate of 25–47% among psychiatric in-patients following restraint,^18^ demonstrating detrimental psychological impacts. Considering the harmful psychological impact of restrictive practices, it is unsurprising that they have been found to hinder patient recovery and increase the length of hospital stay.^15,21^ Likewise, members of staff who have used restrictive methods report predominantly negative psychological consequences, including guilt, self-doubt and feeling that they have failed at their job.^22,23^ Both groups agree that the use of restrictive practices damages the staff–patient relationship.^24–26^ Although qualitative research investigating patients’ and staffs’ experiences of restrictive practices is reasonably well-established, there has yet to be a systematic review of this evidence. The aforementioned systematic review published in 2019 did not examine qualitative literature and subjective experiences of restraint.^18^ Thus, the aim of the present study is to examine patients’ and staff members’ experiences of restrictive practices in acute psychiatric in-patient settings. To understand the idiosyncrasies of the experience between groups and thus make important recommendations, patients’ and staff members’ experiences of restrictive practices will be explored separately, allowing for comparisons to be made.

## Method

A thematic synthesis of the current qualitative literature on patient and staff experiences of restrictive practices in acute mental health in-patient settings was conducted following the method proposed by Thomas & Harden.^27^ The protocol for the current review was pre-registered on PROSPERO (CRD42020176859) and the Preferred Reporting Items for Systematic Reviews and Meta-Analyses (PRISMA) guidance was followed.^28^

### Study eligibility criteria

Studies were included if they (a) had the primary aim of exploring patients’ or staff members’ experiences of restrictive practices in acute psychiatric in-patient settings, (b) adopted a qualitative methodology (e.g. interviews and focus groups) and (c) recruited patients and/or staff members aged 18 years or over who had experience of restrictive practices. Although the motivation is to understand experiences of restrictive practice to inform UK practice, as this review is the first of its kind, an international perspective is warranted. Therefore, no restrictions were placed on location of study. Studies not published in English were excluded to ensure that the studies were fully accessible to the reviewer. No publication date limitations were applied to the search because of concerns about the potential paucity of literature.

### Search strategy and study extraction

The systematic search of three electronic databases (PsycInfo, Embase and MEDLINE) was conducted independently by H.B. in May 2020 and updated in September 2021. These databases were chosen as they are recommended for the identification of nursing journals, as well as allowing the search to extend across the psychological and medical literature. After identifying relevant studies from the initial search, citation searching of key papers was carried out to ensure the maximum number of relevant studies were captured by the review.

The search terms were determined using the SPIDER framework^29^ to ensure that they captured the concepts within the review question. The search terms were developed by drawing on existing reviews’ search terms and the keywords of potential papers of interest. The final list of search terms was developed with assistance from a health librarian and terms were grouped into in-patient terms, staff and patient terms, qualitative terms and restraint terms. The full search strategy can be found in the supplementary material, available at https://doi.org/10.1192/bjo.2022.574. Titles and abstracts were initially searched by H.B. From these, potentially eligible full texts were identified and screened by H.B. Any uncertainties were discussed in supervision with L.W. until a final list of full texts was decided upon.

### Quality assessment

Quality of the studies was assessed using the Critical Appraisal Skills Programme (CASP) tool, which examines the validity, results and relevance of findings. This was to ensure that the studies included were sufficiently rigorous and trustworthy. We chose to use the CASP checklist as it is the most commonly used tool in assessing quality in qualitative literature. The checklist examines whether the data collection processes are reliable and valid, whether the analysis process is robust and whether ethical issues have been fully considered.

### Data extraction and synthesis

Overall, the analysis was led by H.B. and any uncertainties were discussed in supervision with L.W. and S.R. Study characteristics were initially extracted into a pre-defined table by H.B. The data extracted included study characteristics (location, data collection method, method of restraint investigated, sample population) and participant characteristics (gender, ethnicity and, if applicable, diagnosis). The full ‘results’ or ‘findings’ section of the paper were also extracted for analysis.

Guided by the method adopted by Thomas & Harden,^27^ any parts of the studies labelled as ‘results’ or ‘findings’ were included in the analysis. Analysis was conducted using QSR's NVivo 12 software^30^ on macOS, and each included full text was imported into the software. Although the interpretations of study authors contributed to the analysis, direct quotes from patients and staff were prioritised to ensure that the review accurately captured their experiences in the way they were originally reported. Data pertaining to patients and staff members were analysed separately. A critical realist position was taken, which explains that psychological and social constructs exist but the way they are understood is subjective and dependent on the individual's personal experience.^24^ Data were also analysed from an inductive approach. The first step of the analysis involved the free coding of text containing information relevant to the research question. To do this H.B. read and re-read the transcript to ensure familiarisation with the data. Line by line coding was then undertaken and codes were highlighted if they were thought to represent patient or staff experiences of restrictive practices. A code was often represented by a single word or short phrase. Related codes were then organised into descriptive themes, some of which were similar to the themes identified in the original studies. Finally, a second grouping process reorganised descriptive themes into analytical themes and subthemes. These aimed to capture the meaning patients and staff gave to restrictive practices.

## Results

### Study selection

**Fig. 1:**
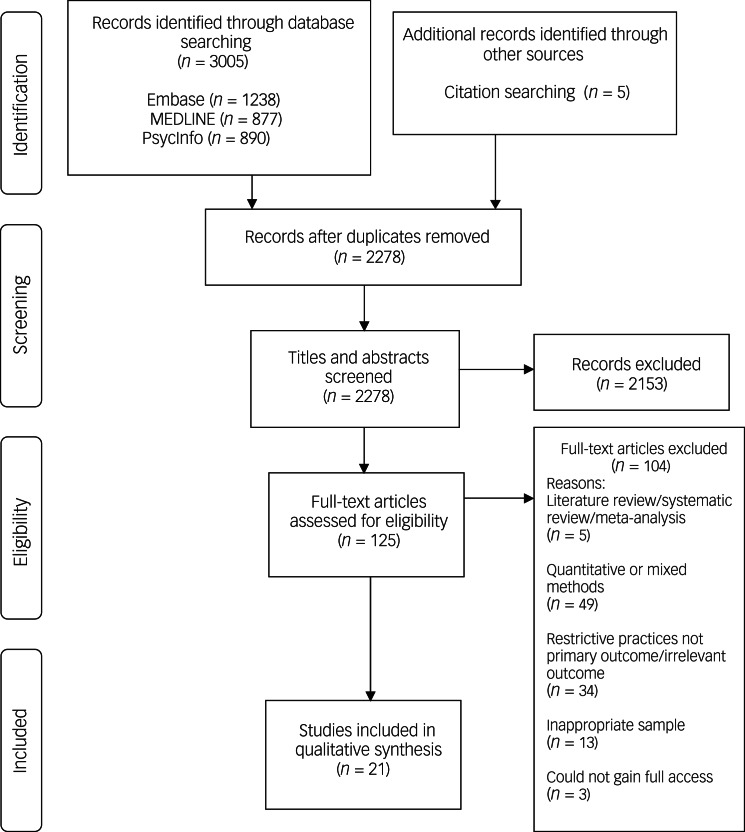
PRISMA diagram.

After duplicates were removed, 2278 studies were identified. After screening the titles and abstracts against the inclusion criteria, 125 full-text articles were identified and these were subsequently screened to determine their inclusion in the current review. A further 104 studies were excluded as they did not meet the inclusion criteria, resulting in 21 studies being included in the synthesis. The study selection process is outlined in Fig. 1.

### Quality assessment

The outcome of the quality assessment using the CASP tool can be found supplementary Table 1. The majority of studies were moderate to high quality and were considered to be sufficiently rigorous in terms of their methodology, design and analysis. There has been much debate about whether it is meaningful to exclude qualitative studies based on assessments of their quality.^31^ In the current review, the synthesis would not have been improved by the exclusion of weaker studies, as their findings were consistent with those from the higher-quality studies.^32^ Therefore, no studies were excluded on the basis of the quality assessment as they were all considered to capture the experiences of patients and staff effectively.

### Study characteristics

The study characteristics for the 21 included papers are summarised in Table 1. The studies had been conducted across 11 countries and were published between 1996 and 2019. They used a range of qualitative data collection methods, including focus groups, interviews, reflective writing and debrief forms. Ten studies focused solely on the experiences of patients, nine investigated the experiences of staff members and two examined the experiences of both groups. The papers covered a range of restrictive practices, including physical restraint, chemical/pharmacological restraint, mechanical restraint, seclusion and deprivation of liberty.

**Table 1:** Summary of study characteristics

Study	Location	Data collection method	Method of restraint investigated	Data analysis method	Sample size	Gender	Participants	Ethnicity
**Patient and staff member studies**								
Bonner et al (2002)^34^	UK	Semi-structured interviews	Manual physical restraint	Huberman's (1984) thematic coding technique	18	n.r.	Mental health in-patients and staff	Unknown
Sequeira & Halstead (2004)^16^	UK	Semi-structured interviews	Physical restraint	Thematic content analysis using grounded theory	36	n.r.	17 nurses in a psychiatric hospital who had restrained patients, the 14 patients who had been restrained, the 5 patients who had observed these events	Unknown
**Patient studies**								
Johnson (1998)^37^	Chicago, USA	Unstructured interviews	Leather restraints	Modified eight-stage interpretative process grounded in Heideggerian phenomenology	10	5 male, 5 female	Psychiatric in-patients	8 Caucasian, 2 African American
Meehan et al (2000)^56^	Queensland, Australia	Semi-structured interviews	Seclusion	Content analysis	12	7 male, 5 female	Acute psychiatric in-patients	Unknown
Sequeira & Halstead (2002)^35^	UK	Semi-structured interviews	Physical restraint	Thematic content analysis using grounded theory	14	n.r.	Psychiatric in-patients with a range of diagnoses, including personality disorder and enduring mental illness	Unknown
Wynn (2004)^20^	Norway	Unstructured interviews	Physical restraint, including pharmacological restraint	Grounded theory	12	n.r.	Psychiatric in-patients (7 paranoid schizophrenia, 3 bipolar disorder, 2 emotionally unstable personality disorder)	Unknown
Chien et al (2005)^33^	Hong Kong	Semi-structured interviews	First experience of physical restraint	Content analysis and coding into themes	30	18 male, 12 female	Psychiatric in-patients	23 Chinese, 7 American Chinese and Asian
Kuosmanen et al (2007)^57^	Finland	Semi-structured interviews	Deprivation of liberty (leaving ward, confiscation of property, coercive measures)	Inductive content analysis	51	25 male, 26 female	In-patients on two acute psychiatric wards	Unknown
Kontio et al (2012)^36^	Southern Finland	Focused interviews	Restraint and seclusion	Inductive content analysis	30	19 male, 11 female	In-patients from six acute psychiatric wards (18 schizophrenia, 7 psychotic mood disorder, 4 drug-related psychosis, 1 other psychosis)	Unknown
Faschingbauer et al (2013)^19^	Mid-western USA	Unstructured interviews	Seclusion	van Manen's (1990) phenomenological approach	12	6 male, 6 female	Psychiatric in-patients with primary diagnoses including bipolar disorder, PTSD, major depression, substance misuse, borderline personality disorder	8 Caucasian, 2 Native American, 2 African American
Ezeobele et al (2014)^38^	South-western USA	Semi-structured interviews	Seclusion	Colaizzi's (1978) phenomenological methodology	20	12 male, 8 female	Psychiatric in-patients with diagnoses including bipolar disorder, paranoid schizophrenia, schizoaffective disorder, major depression, other psychosis	Unknown
Ling et al (2015)^58^	Canada	Restraint debrief forms	Seclusion, chemical and physical restraint	Thematic analysis following guidelines outlined by Braun & Clarke (2006)	55 debrief forms	n.r.	Psychiatric in-patients with diagnoses including anxiety, schizophrenia, early psychosis	Unknown
**Staff member studies**								
Muir-Cochrane (1996)^50^	Australia	Reflective writing and semi-structured interviews	Seclusion	Grounded theory	7	n.r.	Psychiatric nurses working in two closed wards in a teaching hospital	Unknown
Marangos-Frost & Wells (2000)^17^	Toronto, Canada	Structured ethnographic interviews	Physical restraint	Topic coding system and Ethnograph software, following Tesch's 1990 guidelines	6	6 female	Nurses from an in-patient psychiatric unit	Unknown
McCain & Kornegay (2005)^39^	Mid-western United States	Interviews	Behavioural health restraint	Content analysis with Colaizzi's (1978) phenomenological methodology	8	3 male, 5 female	Psychiatric nurses from a psychiatric unit	Unknown
Bigwood & Crowe (2008)^22^	New Zealand	Semi-structured interviews	Physical restraint	van Manen's (1990) descriptive hermeneutic phenomenological approach	7	4 male, 3 female	Mental health nurses based in an acute in-patient psychiatric setting	Unknown
Moran et al (2009)^23^	Ireland	Focus group interviews of 6–9 nurses	Restraint and seclusion	Qualitative interpretive analysis	23	8 male, 15 female	Psychiatric hospital nurses	Unknown
Fereidooni Moghadam et al (2014)^25^	Ahvaz, Southern Iran	Semi-structured interviews	Physical restraint	Inductive content analysis	14	n.r.	Psychiatric nurses across four psychiatric hospitals	Unknown
Muir-Cochrane et al (2015)^26^	Melbourne, Australia	Individual interviews	Restraint and seclusion	Smith and Osborn's (2008) approach to interpretative phenomenological analysis	39	n.r.	Nurses working in three acute old age psychiatric in-patient units	Unknown
Vedana et al (2018)^12^	Brazil	Semi-structured interviews	Physical and mechanical restraint	Thematic analysis and symbolic interactionism	29	15 male, 14 female	Nursing staff from two psychiatric units	Unknown
Haugom et al (2019)^24^	Norway	Semi-structured form	Seclusion	Qualitative content analysis based on Graneheim & Lundman (2004)	149 detailed written descriptions of episodes of seclusion	n.r.	Clinical staff (psychiatrists and psychologists) and ward staff (psychiatric nurses, social educators and social workers) from 57 psychiatric wards	Unknown

n.r., not reported; PTSD, post-traumatic stress disorder.

### Patient synthesis

The four overarching themes (‘the psychological effects’, ‘staff communication’, ‘loss of autonomy’ and ‘making changes’) and their subthemes that emerged from the 12 qualitative studies that explored patients’ experiences are listed in Appendix 1 The studies that contributed to each theme and subtheme relating to patients are outlined supplementary Table 2.

#### Theme 1: The psychological effects

The theme of psychological effects was present in all 12 studies. Patients’ descriptions were predominantly negative. Three subthemes were identified: ‘a distressing experience’, ‘re-traumatisation’ and ‘the aftermath’.

##### A distressing experience

The majority of patients experienced restraint as a psychologically distressing event. Being restrained immediately induced intense emotions of anxiety and fear, which persisted throughout the whole restraint. In 5 of the 12 studies, patients reported that they became so distressed during their experience that they feared for their own life (although in 7 studies, some participants described how restraint made them feel comforted and safe):

‘I was afraid that someone might hurt me suddenly while I was being restrained … I was scared … nobody seemed willing to help me calm this fear and I was afraid the staff were never going to take the restraint off.’^33^

‘I thought they were going to kill me.’^34^

##### Re-traumatisation

Some of the patients described how being restrained reawakened personal traumatic memories of past abuse, such as rape or other violent incidents, suggesting that restraint may cause psychological damage and re-traumatisation. Participants described a wide array of trauma-related psychological experiences:

‘It's scary, and like if they're restraining you to give you an injection, they're undoing your trousers or pulling your skirt off. It kind of reminds me of like my past when I was abused and it really gets to you.’^35^

##### The aftermath

Patients described the persistent psychological impact of restrictive practices as wholly negative. They described persistent post-traumatic-type experiences following a restraint, such as nightmares, intrusive memories and emotional distress:

‘Sometimes me keep dreaming things, having nightmares. Don't know why, I do worry about it.’^35^

#### Theme 2: Staff communication

Patients reported that the quality of staff communication could determine whether a situation escalated to the point of restraint. Communication quality was also described as fundamental in helping them understand why they were being restrained or secluded. The two sub-themes identified within this theme were: ‘inadequate communication’ and ‘effective communication’.

##### Inadequate communication

Patients perceived poor staff communication skills to be one of the most common causes of their behaviour escalating to the point of restraint. Participants described, for example, staff not being responsive, actively listening or being clear about their expectations of the patient. Patients in eight of the studies described the absence of communication during and after the restraint event, for example the lack of a debrief. This led to unresolved emotions and unanswered questions following the event, and feelings that the restraint was unjust:

‘ *…* I didn't understand why they put me into the seclusion room and I never got information on this. The staff was reluctant to provide information on why and how long, what next *…* ’^36^

##### Effective communication

Just 1 of the 12 studies described instances of effective staff communication during restraint. Effective communication was characterised by a calm, clear and transparent approach that explained the restraint process. It is evident from the account how effective staff communication can improve the patient's experience:

‘ … They approached me in a caring and calm manner and explained to me what was happening to me. When I was being restrained, I felt relieved and clearly remembered the moment that the nursing officer explained to my parents in front of me … about why the restraint had been applied and what had been done to me.’^33^

#### Theme 3: Loss of human rights

The experience of losing human rights was reflected in all 12 of the studies. Patients described feeling powerless and helpless, which was frequently perceived to be a consequence of staff actions. Three subthemes emerged: ‘power struggle’, ‘imprisonment’ and ‘dehumanisation’.

##### Power struggle

Patients felt that staff abused their powers by using restrictive practices too readily, leading to a ward culture of ‘them versus us’. Several patients recalled restraint situations where they felt staff were laughing at them, which resulted in the perception that staff were ridiculing them and ultimately against them. Patients’ perception that staff abused their power exacerbated feelings of powerlessness; powerlessness was reported particularly frequently by female patients who were restrained by male members of staff:

‘That almost set me off again, because, you know, these are your nurses, they are supposed to be taking care of you, and you don't feel like you are being taken care of when someone is making fun or laughing at your situation.’^19^

##### Imprisonment

In 10 of the 12 studies, patients likened their experiences of being admitted to hospital to being imprisoned. Participants felt that the hospital procedures were restrictive and unwelcoming rather than caring and compassionate. In particular, they felt that seclusion rooms resembled a prison as their freedom of movement was restricted:

‘The staff wouldn't release me, even for a while, so that I could use the washroom. They brought me a bedpan but did not change the bed sheets for me, even though they were contaminated. They only un-cuffed one hand for me to eat … I couldn't do anything. It was just like being chained up in a prison.’^33^

##### Dehumanisation

For some patients, being restrained or secluded felt dehumanising and like they were being treated like animals. Others described feeling like a slave being controlled by ward staff. It was the loss of freedom and autonomy that particularly contributed to the feeling of dehumanisation. Moreover, accounts of restraint and seclusion highlighted distinct care failures and some patients felt that their basic needs were neglected by staff:

‘I felt very uncomfortable. Like I was an animal being chained up. Only difference was, wasn't chains around my neck … I felt dirty … Not being funny, but, my mind went back to stories my grandma told me about slavery days. I felt like I was a slave. I was chained up; I couldn't do anything. I was under somebody else's command … ’^37^

#### Theme 4: Making changes

The theme of making changes emerged from the negative experiences reported by patients, the inadequacies and faults they identified and the suggestions they put forward to remedy these. Three subthemes describe these: ‘preventing restrictive practices’, ‘improving restrictive practices’ and ‘alternatives to restrictive practices’.

##### Preventing restrictive practices

Patients frequently attributed their behaviour escalation and subsequent restraint to lack of communication from staff, for example about ward processes and procedures and what was expected of them. Patients reported that staff also did not talk to them in a compassionate or calm manner, which they explained further aggravated the situation. They suggested that staff should be given training on how to communicate in a way that may prevent behaviour escalating:

‘ … if staff would have talked to me and say ah … you are not supposed to do that … instead of yelling at me like a child … the staff should listen before responding … they need to learn how to talk to patients with respect … learn how to communicate with us all over again … ’^38^

Patients believed that they would be able to use their own coping strategies to avoid restraint if staff prompted them to do so before their behaviour escalated to a certain level. Being allowed to talk to peers on the ward and strategies learned from anger management classes were identified as potential coping mechanisms.

One patient expressed how gaining an understanding of why they were restrained may prevent them from being restrained in the future:

‘The nurses have sat down with me and gone through my records with me. It feels better to look back with a better insight. You can see where you were going wrong. You can see where to make changes … ’^34^

##### Improving restrictive practices

Many patients viewed their treatment while in restraint or seclusion as dehumanising and a violation of their rights. Three papers reported patients’ views of how restrictive practices could be improved. One patient called for more humane treatment:

‘ … I hope that I am a human being in a psychiatric hospital and in the seclusion room too. I want polite, humane behaviour from the staff … ’^36^

Patients also suggested that the introduction of colour, soft furniture and relaxing music in the seclusion rooms could make seclusion a less unpleasant experience.

##### Alternatives to restrictive practices

Patients offered several alternatives to the use of restrictive practices, including one-to-one observation, medication, being left alone in their room, time-out programmes, psychiatric intensive care units and being provided with an activity such as a book to read. The most common suggestion was that open communication and discussion would be the most effective alternative:

‘ … It is essential to try to solve the difficult situation by discussion instead of using coercion (e.g. seclusion room) … ’^36^

### Staff synthesis

The four primary themes (‘the need for restrictive practices’, ‘the psychological impact’, ‘decision-making’ and ‘making changes’) and their subthemes that emerged from the 11 qualitative studies exploring staff experiences are listed in Appendix 2. The studies that contributed to each theme and subtheme relating to staff are outlined supplementary Table 3.

#### Theme 1: The need for restrictive practices

This theme was identified in 9 of the 11 studies. The most common attitudes towards whether restrictive practices are necessary are reflected in the three subthemes: staff members perceived the use of restrictive practices to be ‘the last resort’ after trying alternatives with the aim of ‘avoiding restraint’. However, the most apparent view was that ‘restraint is unavoidable’.

##### The last resort

It was evident that the majority of staff members dreaded having to use restraint and were aware of the power they had and did not want to abuse this power. They described their willingness to try several less restrictive alternatives before resorting to restraint:

‘Restraint and seclusion … is the last resort option; we make a lot of decisions about options before we seclude.’^26^

‘We are aware that we have some power in our job and it is absolutely necessary that we do not abuse it.’^24^

##### Avoiding restraint

Staff described the less restrictive methods they attempted to use before employing restrictive options. These included moving patients to low-stimulus areas, reducing risk, giving them a choice about how the situation was managed, talking through their emotions and medication. Staff were pleased if they were able to successfully avoid using restraint:

‘ … we have good experience avoiding the use of mechanical restraint with this patient, using female staff, spending enough time and being patient.’^24^

‘I do enjoy getting the opportunity to use skills to de-escalate a situation and the majority of times you do.’^22^

##### Restraint is unavoidable

Despite the majority of staff endorsing the use of less restrictive alternatives, the most prevalent attitude was that restrictive practices were unavoidable. The studies suggested that staff believed restrictive practices were the only guaranteed way of preventing harm on the ward, and many accepted it as an essential part of the job:

‘It is necessary in controlling them [patients] … for the time being, it's the only thing to protect the staff and other clients.’^26^

‘I can't see any way around it … I just don't know what else we could do.’^22^

#### Theme 2: The psychological impact

The psychological impact of restraint use was explored in every study. Administering restrictive measures was a predominantly negative experience for staff, and many described the psychological consequences. Three subthemes emerged: ‘distress’, ‘mental conflict’ and ‘nobody to talk to’.

##### Distress

Staff disliked using restrictive practices as they considered them to be distressing and unpleasant for everyone involved. Staff frequently reported anxiety, fear and sadness when asked about their experiences. These feelings persisted post-restraint:

‘Well, it [restraining a patient] certainly would not have a good feeling; nobody likes to restrain another person. Anyway, he/she is a human being, restraining someone else or any living creature does not have a good feeling; definitely it doesn't create a good effect.’^25^

‘ … you go home with a horrible feeling. You have to really work hard at turning off all these horrible feelings.’^16^

##### Mental conflict

Staff members felt that using restraint did not fit with their job role. This was particularly true for nursing staff, who felt conflicted when employing methods that they did not perceive to be caring or therapeutic. This often resulted in feelings of guilt:

‘I wanted to be a nurse . . . but seclusion . . . it's against your nursing principles, it's against your caring attitude … ’^23^

‘I felt instantly like a bully. I felt instantly like, I am awful, you know, look what I have done to this man … ’^22^

##### Nobody to talk to

Staff reported a distinct lack of support from management following their involvement in a restraint event. One individual described the difficulty in finding someone to talk to who would understand:

‘And the other thing of course about this kind of situation [restraint] is that there is nobody you can talk to about that apart from other psychiatric nurses. This essential part of your job you cannot discuss with anyone else because they just can't understand it.’^22^

#### Theme 3: Decision-making

Several factors that contributed to staff members’ decisions to employ restrictive practices were identified in the studies. The three main factors were: ‘risk assessment’, ‘availability of staff’ and ‘availability of alternatives’.

##### Risk assessment

If staff deemed the situation to be high risk to the patient, others in the vicinity or themselves, restrictive practices more likely to be used:

‘Physical restraints are necessary at some point, but they are really to prevent harm to self or others.’^39^

##### Availability of staff

The studies suggested that the availability and competency of staff was the second most important factor considered when deciding whether to use restrictive measures. A number of staff members experienced stress and frustration when having someone restrained on the ward because it often left them understaffed:

‘Patients in restraints go on constant observation and we lose a staff off the floor until we can call and get somebody to come in. But we are still losing someone for an hour or an hour and a half. And sometimes there is nobody available to come in, and in that case we just kind of rotate through. So it makes for a lot heavier workload for everybody when we put someone in restraints.’^17^

##### Availability of alternatives

Members of staff reported restraint to be reasonably uncommon owing to successful preventive measures:

‘You probably only have to restrain, I don't know, 1 in 20 incidents that you actually go to … The vast majority of times we talk the person down, you know.’^22^

However, some believed that there were often no other alternatives available that would have adequately protected everyone from harm in the way that restraint could:

‘ … it needs to be here unless you replace restraint with something that is truly effective.’^12^

#### Theme 4: Making changes

Members of staff voiced dissatisfaction towards employing restrictive practices and worried about the psychological and physical harm that may be caused to the patient, others in the vicinity and staff themselves. Two subthemes were identified from staff suggestions regarding how to improve and reduce restrictive practices: ‘somebody to talk to’ and ‘a safer alternative’.

##### Somebody to talk to

Being involved in the use of restrictive practices was unpleasant and distressing for most staff members. They felt they were inadequately supported following a restraint, and those who were able to talk to someone following the event found it to be unhelpful. One member of staff described how having a supportive person to talk to after an incident was beneficial:

‘You are always thinking “Could I have done something before the event or caught it before we had to do that?”, and I think that once you can sit down and talk that reassures you when you've had that chance. Debriefing, even if it's only in the form of a cup of tea. Nothing major, but still useful. You never know how it's going to go and it's helpful to look back over it to see if you have done everything that you should. After support. I have very strong feelings about this … ’^34^

Some proposed that patients should also have access to support following the event and felt that this could help repair any damage to the staff–patient relationship:

‘They want to talk about things afterwards and I think it can damage the relationship if you don't. Patients don't want someone just to come along, shout orders or whatever, or grab them without then going back and explaining or talking it through. You can end up with a lot of resentment.’^34^

##### A safer alternative

Members of staff called for an effective and safer alternative to restrictive methods. Some preferred the use of relaxation techniques, counselling or constant monitoring. However, this was very much dependent on availability and staffing levels. The studies also suggested that staff perceived restraint to be the only effective option when trying to control high-risk behaviour:

‘It is necessary in controlling them [patients] … for the time being, it's the only thing to protect the staff and other clients.’^26^

## Discussion

The experiences of restrictive practices in psychiatric in-patient settings were overwhelmingly negative for both patients and staff, according to the 21 papers included in this thematic synthesis. Patients described experiencing intense feelings of fear, powerlessness and humiliation while being restrained or secluded. Moreover, patients who had past experiences of physical or sexual abuse described being re-traumatised. Although this arose in only 4 of the 12 studies exploring patients’ experiences, it has important implications considering that a large proportion of female patients in psychiatric hospitals have reported being victims of severe physical or sexual abuse.^40^ The psychological consequences reported by patients appear to be entirely conflictual with the safe, humane and therapeutic environment that is supposed to be provided by in-patient care.^41^ Psychological distress was also a dominant theme within staff experiences of restrictive practices. Importantly, for both patients and staff, the negative emotional consequences persisted after the restraint event was over, suggesting that experiencing or implementing restrictive practices may have long-term psychological consequences. The long-term psychological consequences of experiencing or implementing restrictive practices is something that future research should address.

Patients and staff offered several suggestions for improving restrictive measures (Appendix 3). Similarly, both patients and staff identified the importance of support following the use of a restrictive measure such as restraint or seclusion. Patients felt ignored and neglected during restraint or seclusion, which intensified the distress experienced during the event. Following the event, patients reported receiving no emotional support or explanation and were left with resentment towards staff, which damaged the patient–staff relationship. A recent report from the UK Care Quality Commission highlighted that staffing and time pressures are a hindrance to staff being able to spend time building relationships with patients.^42^ Interestingly, where staff were unable to spend time providing emotional support to patients, patients perceived this as being ignored, leading to increased tensions and a disconnect between patients and staff. The tensions in the staff–patient therapeutic relationship and the importance of having an appropriate ratio of patients to staff has been identified in other international in-patient contexts.^43^ This disconnect could be addressed through strategies such as the application of the Safewards model, which has been successful in decreasing conflict by 15% and containment by 26.4% on a number of psychiatric wards.^44^ Safewards is an organisational approach to delivering in-patient services that aims to minimise the use of restrictive practices through the use of a variety of interventions that promote a therapeutic response, minimise conflict and increase patient safety.^44^ Another potential model is the Six Core Strategies to Reduce the Use of Seclusion and Restraint, a US model that focuses on organisational and leadership change, workforce development, having patient roles such as peer support workers in the in-patient setting, and improving debrief techniques, which has also been shown to reduce restrictive practices.^45^

For staff, both occupational and emotional support was lacking. Future programmes seeking to reduce the use of restrictive practices should consider the provision of training covering these, such as in the ReSTRAIN YOURSELF programme.^46^ The ReSTRAIN YOURSELF programme aims to improve patient safety and avoid unnecessary harm by using restraint-reducing approaches.^42^ The absence of support was also apparent in staff members’ accounts of the time following an event. Worryingly, there is a significant relationship between a lack of support and high level of burnout, which has been found to increase depersonalisation, reduce the quality of care provided and increase staff turnover rates.^47–49^ This has problematic implications for psychiatric in-patient care, as these factors may increase the likelihood that restrictive practices will be used and exacerbate the understaffing pressures that in-patient teams already face.^17,50^ It is imperative that staff are adequately debriefed following their involvement in the implementation of restrictive practices, as research suggests that appropriate support measures have the potential to buffer the negative impact of psychological stress for healthcare professionals.^51^

It was apparent that both patients and staff would prefer the use of less restrictive alternatives. Patients pictured this to be in the form of a strong therapeutic relationship, improved open communication, transparency and responsiveness. De-escalation strategies and the provision of therapeutic environments are some examples of interventions that have been implemented.^44^ However, they have not eliminated the need for restrictive practices and further work needs to be undertaken. It is interesting that several patients internalised the cause of the restraint or seclusion after they understood why it had happened. Their accounts suggest that once they gained this understanding, they were able to take responsibility for their actions and think about how they could change their behaviour to avoid the situation happening again. Staff should aim to be transparent with patients about why they were restrained or secluded, as this in itself may be effective in preventing the need for restrictive practices. However, it must be acknowledged that staff believed there were no other alternatives that were as effective at keeping everyone safe from harm once situations had escalated and become risky. In light of this, there must be a continued effort towards making the implementation of restrictive measures safer by ensuring that all members of staff have the skills to use these methods safely. To further minimise the negative effects of restrictive measures, we must shift some of our focus to improving the quality of the support provided to patients before, during and after restraint or seclusion.

### Strengths and limitations

This review is the first thematic synthesis of qualitative research investigating patient and staff experiences of restrictive practices in psychiatric in-patient settings. Synthesising this qualitative research allowed us to present the complex experiences of patients and staff in a way that is truly representative of their original accounts.^27,52^ We also included studies from a variety of geographical regions. Although this is a strength, as it gives our review an international perspective, it is also drawing together literature from quite diverse contexts. For example, medication regimes vary to a large extent across countries, as do the laws determining their use, and therefore some of the qualitative findings may not be fully relevant in the UK. Another example is that one study examined the use of leather restraints,^37^ which is not current practice in the UK. Therefore, overall results should be interpreted tentatively. It is important to note that 18 of the 21 studies did not report the ethnicity of the participants. Individuals from Black and minority ethnic (BME) communities are more likely to be detained under the Mental Health Act 1983, as well as being more likely to be restrained and secluded, and are significantly underrepresented in mental health research.^53^ Future research should prioritise investigating the experiences of BME communities, as well as ensuring that they consider how ethnicity may affect the subjective experience of restrictive practices. The use of three databases and citation searching ensured that the search was adequately comprehensive.^54^ However, only one reviewer screened the studies identified by the search, which may have introduced systematic or random errors to the process of identifying relevant studies.^55^ The validity of this review may also be affected by the 23-year gap between the earliest and most recent study. The use of restrictive practices has changed over this period, with the reduction of restrictive practices becoming a priority.^14^ The experiences of those in the earlier studies may be significantly different from those in more recent studies. Future reviews should limit the search to ensure that only contemporary research relevant to the current in-patient context is included.

### Relevance for clinical practice

This review has identified that both staff and patients find restrictive practices a negative and distressing experience. There is a need to find alternative options to prevent the use of restrictive practices. Existing interventions such as ReSTRAIN YOURSELF may be helpful in addressing these issues, but consistent implementation is a challenge. Both parties acknowledged a need for restrictive practices, and further specialist training and supervision are required to ensure these are implemented safely. Psychological distress was also a dominant theme within patient and staff experiences of restrictive practices. There is a need to provide appropriate reflective and emotional support for patients and staff to manage the effects of restrictive practices.

## Data Availability

Data availability is not applicable to this article as no new data were created or analysed in this study.

## Supplementary material

For supplementary material accompanying this paper visit http://doi.org/10.1192/bjo.2022.574. click here to view supplementary material

## Appendices

### Appendix 1 Patient themes and subthemes

Theme 1: The psychological effects

- A distressing experience
- Re-traumatisation
- The aftermath

Theme 2: Staff communication

- Inadequate communication
- Effective communication

Theme 3: Loss of human rights

- Power struggle
- Imprisonment
- Dehumanisation

Theme 4: Making changes

- Communication and debrief
- Preventing restrictive practices
- Improving restrictive practices
- Alternatives to restrictive practices

### Appendix 2 Staff themes and subthemes

Theme 1: The need for restrictive practices

- The last resort
- Avoiding restraint
- Restraint is unavoidable

Theme 2: The psychological impact

- Distress
- Mental conflict
- Nobody to talk to

Theme 3: Decision-making

- Risk assessment
- Availability of staff
- Availability of alternatives

Theme 4: Making changes

- Somebody to talk to
- A safer alternative

### Appendix 3 Summary of suggested improvements made by patients and staff

#### Patients’ suggestions

Before:

- More clarity from staff on their expectations of patients, including ward procedures and rules
- More training in de-escalation techniques for staff
- Anger management classes for patients

During:

- Open communication about the restraint or seclusion process
- Colour, soft furniture and relaxing music in seclusion

After:

- Debrief/support post-incident

#### Staff members’ suggestions

Before:

- Training in the safe implementation of restrictive measures

After:

- Debrief/support post-incident
