# Supplementary material for: Patients’ and staff members’ experiences of restrictive practices in acute mental health in-patient settings: systematic review and thematic synthesis
Source: BJPsych Open. 2022 Oct 6;8(6):e178. doi: 10.1192/bjo.2022.574 (PMC9634587; doi:10.1192/bjo.2022.574)
Supplement: Supplementary file 1 [file S2056472422005749sup001.docx]

**Supplementary materials**

**Search terms**

‘acute hospital*’ OR 'mental hospital*’ OR ‘psych* hospital*’ OR ‘acute in$patient’ OR ‘mental health in$patient’ OR ‘psych* in$patient’ OR ‘psych* patient*’

AND

‘service user*’ OR ‘*patient*’ OR ‘staff*’ OR ‘worker*’ OR ‘nurs*’ OR ‘doctor*’

AND

‘experience*’ OR ‘account*’ OR ‘observation*’ OR ‘focus group*’ OR ‘diar*’ OR ‘interview*’ OR ‘qualitative’ OR ‘perceptions*’ OR ‘perspective*’ OR ‘thought*’ OR ‘feeling*’ OR ‘understanding’

AND

‘restrain*’ OR ‘sedat*’ OR ‘seclu*’ OR ‘rapid tranquilisation*’ OR ‘blanket ban*’ OR ‘segregat*’ OR ‘restrict*’.

| Table S1 Quality assessment of studies using the Critical Appraisal Skills Programme (CASP) | | | | | | | | | | |
| --- | --- | --- | --- | --- | --- | --- | --- | --- | --- | --- |
| Studies | CASP quality criteria met* | | | | | | | | |  |
|  | **1** | **2** | **3** | **4** | **5** | **6** | **7** | **8** | **9** | **10** |
| Muir-Cochrane, 1996 | ✓ | ✓ | ✓ | ✓ | ✓ | ⨉ | ⨉ | ✓ | ✓ | ✓ |
| Johnson, 1998 | ✓ | ✓ | ✓ | ⨉ | ✓ | ⨉ | ⨉ | ✓ | ✓ | ✓ |
| Marangos-Frost & Wells, 2000 | ✓ | ✓ | ✓ | ⨉ | ✓ | ✓ | ✓ | ✓ | ✓ | ✓ |
| Meehan et al. 2000 | ✓ | ✓ | ✓ | ✓ | ✓ | ✓ | ✓ | ✓ | ✓ | ✓ |
| Bonner et al. 2002 | ✓ | ✓ | ✓ | ✓ | ✓ | ✓ | ✓ | ✓ | ✓ | ✓ |
| Sequeira & Halstead, 2002 | ✓ | ✓ | ✓ | ✓ | ✓ | ✓ | ✓ | ✓ | ✓ | ✓ |
| Sequeira & Halstead, 2004 | ✓ | ✓ | ✓ | ✓ | ✓ | ⨉ | ⨉ | ✓ | ✓ | ✓ |
| Wynn, 2004 | ✓ | ✓ | ✓ | ✓ | ✓ | ⨉ | ✓ | ✓ | ✓ | ✓ |
| Chien et al. 2005 | ✓ | ✓ | ✓ | ✓ | ✓ | ✓ | ✓ | ✓ | ✓ | ✓ |
| McCain & Kornegay, 2005 | ✓ | ✓ | ✓ | ⨉ | ✓ | ⨉ | ✓ | ✓ | ✓ | ✓ |
| Kuosmanen et al. 2007 | ✓ | ✓ | ✓ | ✓ | ✓ | ⨉ | ✓ | ✓ | ✓ | ✓ |
| Bigwood & Crowe, 2008 | ✓ | ✓ | ✓ | ✓ | ✓ | ✓ | ✓ | ✓ | ✓ | ✓ |
| Moran et al. 2009 | ✓ | ✓ | ✓ | ✓ | ✓ | ✓ | ✓ | ✓ | ✓ | ✓ |
| Kontio et al. 2012 | ✓ | ✓ | ✓ | ✓ | ✓ | ✓ | ✓ | ✓ | ✓ | ✓ |
| Faschingbauer et al. 2013 | ✓ | ✓ | ✓ | ⨉ | ✓ | ⨉ | ✓ | ✓ | ✓ | ✓ |
| Ezeobele et al. 2014 | ✓ | ✓ | ✓ | ✓ | ✓ | ✓ | ✓ | ✓ | ✓ | ✓ |
| Fereidooni Moghadam et al. 2014 | ✓ | ✓ | ✓ | ✓ | ✓ | ⨉ | ✓ | ✓ | ✓ | ✓ |
| Ling et al. 2015 | ✓ | ✓ | ✓ | ✓ | ✓ | ⨉ | ⨉ | ✓ | ✓ | ✓ |
| Muir-Cochrane et al. 2015 | ✓ | ✓ | ✓ | ✓ | ✓ | ⨉ | ✓ | ✓ | ✓ | ✓ |
| Vedana et al. 2018 | ✓ | ✓ | ✓ | ✓ | ✓ | ✓ | ✓ | ✓ | ✓ | ✓ |
| Haugom et al. 2019 | ✓ | ✓ | ✓ | ✓ | ✓ | ✓ | ✓ | ✓ | ✓ | ✓ |

*Symbols: ✓ = yes, ⨉ = no/unclear. Criteria: (1) clear aims, (2) appropriate methodology, (3) appropriate research design, (4) appropriate recruitment strategy, (5) data collected in a way that addressed research aims, (6) consideration of researcher-participant relationship, (7) consideration of ethical issues, (8) data analysis sufficiently rigorous, (9) clear findings, (10) consideration of research value.

| Table S2 Service user studies contributing to each theme and subtheme | | | | | | | | | | | | |
| --- | --- | --- | --- | --- | --- | --- | --- | --- | --- | --- | --- | --- |
| Themes and sub-themes | Studies | | | | | | | | | | | |
|  | Johnson, 1998 | Meehan et al. 2000 | Bonner et al. 2002 | Sequeira & Halstead, 2002 | Sequeira & Halstead, 2004 | Wynn, 2004 | Chien et al. 2005 | Kuosmanen et al. 2007 | Kontio et al. 2012 | Faschingbauer et al. 2013 | Ezeobele et al. 2014 | Ling et al. 2015 |
| Theme 1: The psychological effects | | | | | | | | | | | | |
| A distressing experience | ✓ | ✓ | ✓ | ✓ | ✓ | ✓ | ✓ | ✓ | ✓ | ✓ | ✓ | ✓ |
| Re-traumatisation |  |  | ✓ | ✓ |  | ✓ |  |  |  |  | ✓ |  |
| The aftermath |  | ✓ | ✓ | ✓ | ✓ | ✓ | ✓ |  |  | ✓ | ✓ | ✓ |
| Theme 2: Staff communication | | | | | | | | | | | | |
| Inadequate communication | ✓ | ✓ | ✓ | ✓ |  | ✓ | ✓ | ✓ | ✓ | ✓ | ✓ | ✓ |
| Effective communication |  |  |  |  |  |  | ✓ |  |  |  |  |  |
| Theme 3: Loss of human rights | | | | | | | | | | | | |
| Power struggle | ✓ | ✓ | ✓ | ✓ | ✓ | ✓ | ✓ | ✓ | ✓ | ✓ | ✓ | ✓ |
| Imprisonment | ✓ | ✓ |  | ✓ |  | ✓ | ✓ | ✓ | ✓ | ✓ | ✓ | ✓ |
| Dehumanisation | ✓ | ✓ | ✓ | ✓ | ✓ | ✓ | ✓ | ✓ | ✓ | ✓ | ✓ | ✓ |
| Theme 4: Making changes | | | | | | | | | | | | |
| Communication and debrief |  | ✓ | ✓ |  |  |  | ✓ | ✓ | ✓ | ✓ | ✓ |  |
| Preventing restrictive practices |  | ✓ |  |  |  |  |  |  |  | ✓ | ✓ | ✓ |
| Improving restrictive practices |  | ✓ |  |  |  |  |  |  | ✓ |  |  | ✓ |
| Alternatives to restrictive practices |  | ✓ |  |  |  | ✓ |  |  | ✓ | ✓ |  | ✓ |

| Table S3 Staff member studies contributing to each theme and sub-theme | | | | | | | | | | | |
| --- | --- | --- | --- | --- | --- | --- | --- | --- | --- | --- | --- |
| Themes and sub-themes | Studies | | | | | | | | | | |
|  | Muir-Cochrane, 1996 | Marangos-Frost & Wells, 2000 | Bonner et al. 2002 | Sequeira & Halstead, 2004 | McCain & Kornegay, 2005 | Bigwood & Crowe, 2008 | Moran et al. 2009 | Fereidooni Moghadam et al. 2014 | Muir-Cochrane et al. 2015 | Vedana et al. 2018 | Haugom et al. 2019 |
| Theme 1: The need for restrictive practices | | | | | | | | | | | |
| The last resort | ✓ | ✓ | ✓ |  | ✓ |  | ✓ |  | ✓ | ✓ | ✓ |
| Avoiding restraint | ✓ | ✓ |  |  | ✓ | ✓ | ✓ |  |  | ✓ | ✓ |
| Restraint is unavoidable | ✓ | ✓ |  |  | ✓ | ✓ |  |  | ✓ | ✓ |  |
| Theme 2: The psychological impact | | | | | | | | | | | |
| Distress |  | ✓ | ✓ | ✓ | ✓ | ✓ | ✓ | ✓ | ✓ | ✓ |  |
| Mental conflict |  | ✓ | ✓ | ✓ |  | ✓ | ✓ |  | ✓ | ✓ | ✓ |
| Nobody to talk to |  |  | ✓ | ✓ |  | ✓ | ✓ |  |  |  | ✓ |
| Theme 3: Decision making | | | | | | | | | | | |
| Risk assessment | ✓ | ✓ | ✓ | ✓ | ✓ | ✓ |  | ✓ | ✓ | ✓ | ✓ |
| Availability of staff | ✓ | ✓ |  |  |  |  | ✓ |  | ✓ | ✓ | ✓ |
| Availability of alternatives | ✓ | ✓ |  |  |  |  |  |  |  |  |  |
| Theme 4: Making changes | | | | | | | | | | | |
| Somebody to talk to |  |  | ✓ | ✓ |  | ✓ |  |  |  | ✓ | ✓ |
| A safer alternative |  |  |  | ✓ | ✓ |  |  |  | ✓ | ✓ | ✓ |
